# Evaluation of Daily Physical Activity (DPA) policy implementation in Ontario: surveys of elementary school administrators and teachers

**DOI:** 10.1186/s12889-016-3423-0

**Published:** 2016-08-08

**Authors:** Kenneth R. Allison, Karen Vu-Nguyen, Bessie Ng, Nour Schoueri-Mychasiw, John J. M. Dwyer, Heather Manson, Erin Hobin, Steve Manske, Jennifer Robertson

**Affiliations:** 1 Public Health Ontario, 480 University Avenue, Suite 300, Toronto, ON M5G 1V2 Canada; 2 Department of Family Relations and Applied Nutrition, University of Guelph, Macdonald Institute Building, 50 Stone Road East, Guelph, ON Canada N1G 2W1; 3 Propel Centre for Population Health Impact, University of Waterloo, Waterloo, ON N2L 3G1 Canada

**Keywords:** Daily physical activity, Policy, Evaluation, Implementation, Fidelity, School, Surveys, Administrators, Teachers

## Abstract

**Background:**

School-based structured opportunities for physical activity can provide health-related benefits to children and youth, and contribute to international guidelines recommending 60 min of moderate-to-vigorous physical activity (MVPA) per day. In 2005, the Ministry of Education in Ontario, Canada, released the Daily Physical Activity (DPA) policy requiring school boards to “ensure that all elementary students, including students with special needs, have a minimum of twenty minutes of sustained MVPA each school day during instructional time”. This paper reports on the first provincial study evaluating implementation fidelity to the DPA policy in Ontario elementary schools and classrooms. Using an adapted conceptual framework, the study also examined associations between implementation of DPA and a number of predictors in each of these respective settings.

**Methods:**

Separate cross-sectional online surveys were conducted in 2014 with Ontario elementary school administrators and classroom teachers, based on a representative random sample of schools and classrooms. An implementation fidelity score was developed based on six required components of the DPA policy. Other survey items measured potential predictors of implementation at the school and classroom levels. Descriptive analyses included frequency distributions of implementation fidelity and predictor variables. Bivariate analyses examining associations between implementation and predictors included binary logistic regression for school level data and generalized linear mixed models for classroom level data, in order to adjust for school-level clustering effects.

**Results:**

Among administrators, 61.4 % reported implementation fidelity to the policy at the school level, while 50.0 % of teachers reported fidelity at the classroom level. Several factors were found to be significantly associated with implementation fidelity in both school and classroom settings including: awareness of policy requirements; scheduling; monitoring; use of resources and supports; perception that the policy is realistic and achievable; and specific barriers to implementation.

**Conclusions:**

Findings from the surveys indicate incomplete policy implementation and a number of factors significantly associated with implementation fidelity. The results indicate a number of important implications for policy, practice and further research, including the need for additional research to monitor implementation and its predictors, and assess the impacts of study recommendations and subsequent outcomes of a reinvigorated DPA moving forward.

## Background

### Physical activity benefits and trends

Regular moderate-to-vigorous physical activity (MVPA) by children and youth contributes to their physical (e.g., adiposity, skeletal health, cardiorespiratory fitness) and mental health [1–5]. Consistent with international physical activity guidelines [6], Canadian guidelines recommend that children and youth should accumulate at least 60 min of MVPA per day for health benefits [7]. However, recent data based on direct measures of physical activity indicated that only 9 % of Canadian children and youth (age 5-17) are meeting that guideline [8]. Moreover, it is widely recognized that there are consistent declines in physical activity, especially among females, as students progress into and through secondary school [9].

### School-based opportunities for physical activity

Provision of school based opportunities for physical activity is important since physical education and physical activity policies and programs are associated with improved physical activity, fitness, and other health outcomes among students [10–13]. Also, school-based physical activity is associated with improved classroom behaviour, concentration, and academic performance [14–16].

Traditionally, physical education (PE) classes and recess have provided elementary school students the venue and time for structured and unstructured physical activity during the school day. However, PE and recess vary across and within jurisdictions (states, provinces, school boards), leading to differences in the amount of physical activity that students receive each day [17, 18]. In many cases, PE class does not meet every school day and sometimes the class is devoted to health subjects in a classroom setting. For example, in Ontario, PE is a component of the health and physical education (HPE) curriculum. Similarly, student activity levels during recess can vary [14].

In response to the need for increased physical activity, other school-based opportunities have been developed to supplement traditional PE class and recess [14]. In 2005, the Ontario Ministry of Education (EDU) released Policy/Program Memorandum (PPM) No. 138: Daily Physical Activity (DPA) in elementary schools, Grades 1–8. This policy requires publicly funded school boards to “ensure that all elementary students (grades 1–8), including students with special needs, have a minimum of twenty minutes of sustained MVPA each school day during instructional time” [19]. DPA was intended to be offered on days in which PE class was not scheduled or when students did not receive this amount of physical activity during the time allotted for PE class (i.e., when health subjects were being taught).

Full implementation of the DPA policy was projected for the end of the 2005-06 school year. Based on a retrospective analysis of the DPA policy development and initial implementation, several factors were central to its acceptance in the education system. These factors included the need for flexibility in planning, scheduling and delivering DPA sessions at the school board, school, and classroom levels [20].

Various DPA initiatives have been developed and implemented in other parts of Canada as well [21–24]. A unique feature of Ontario’s DPA policy, compared to similar policies or programs in other provinces, is that it must be offered during instructional time – not during recess, lunch hour, or after school. As such, it is a required component of the curriculum as well as a provincial policy.

### Factors affecting implementation of school-based physical activity interventions

Many types of school-based strategies for increasing student physical activity, such as DPA, have been implemented throughout Canada and other countries with varied success [14, 24]. In addition to differences in strategies, various contextual factors can influence the implementation of policies or curricula. Consistent with the Social Ecological Model [25], many of these factors can be conceptualized at the organizational, interpersonal, and individual levels.

*Organizational-level factors* found to affect successful implementation of DPA or other physical activity/education interventions in schools include the provision of appropriate training and resources to teachers [26–28], having a physical education specialist teacher working in the school [29–31], availability of time within the curriculum [28, 30, 32, 33], space/facilities [28, 30, 34, 35], equipment [27, 36, 37], and budget [29, 34]. Lack of accountability and performance measures required for the program have also been identified as barriers to successful implementation of physical activity interventions [36, 38].

*Interpersonal-level factors* affecting implementation that school administrators and teachers may experience include the level at which the program is seen to be supported and prioritized within the school environment [27, 28, 32, 37, 39], and by community partnerships [26, 37, 40] and parents [26, 29, 37].

At the *individual teacher level*, some factors that may influence implementation include the teacher’s personality [39], self-efficacy/confidence level [27, 30, 39], relevant education, and experience with physical activity [28, 31, 39]*.* Teacher’s beliefs about the importance of the program may also affect implementation [27, 29, 33]*.*

### Existing studies of DPA implementation in Ontario

A number of previous studies have assessed components of DPA in Ontario. Robertson-Wilson and Lévesque examined DPA’s fit with the Hogwood and Gunn preconditions for perfect policy implementation and found that several preconditions were accounted for in the development of the policy [41]. However, the strategy had remaining gaps that could be addressed to facilitate optimal implementation, including resource sustainability, perceived value of the policy, and evaluation plans [41].

Stone and colleagues conducted an assessment in Greater Toronto Area schools as to whether DPA was meeting its objectives in reach (all students), duration (20 min), intensity (moderate to vigorous) and frequency (every day) of physical activity [42]. Using accelerometers, the study found that most schools were not sufficiently implementing DPA, especially the requirement of 20 min of continuous MVPA [42]. Also, Patton conducted a survey of teachers’ perspectives and experiences in implementing DPA in the Thames Valley District School Board (London, Ontario) [38]. Study findings indicated that DPA was not being conducted as intended in terms of duration, intensity, or frequency. Respondents also reported a number of barriers to program delivery [38]*.*

The findings from these previous studies contribute to our understanding of the current levels of DPA policy implementation and the important factors associated with it; however, none of these studies provided a provincial-level assessment of the status of DPA implementation. In 2012, a joint report by Cancer Care Ontario and Public Health Ontario (PHO) made an evidence-informed recommendation to the Ontario government calling for the evaluation of the status and quality of DPA in Ontario elementary schools [43]. Such an evaluation was considered a means of supporting government accountability for monitoring this policy initiative and establishing a process for assessing intervention quality. To address this gap, the authors initiated related studies to assess DPA policy implementation on a provincial level.

### Conceptual framework for the study

**Fig. 1:**
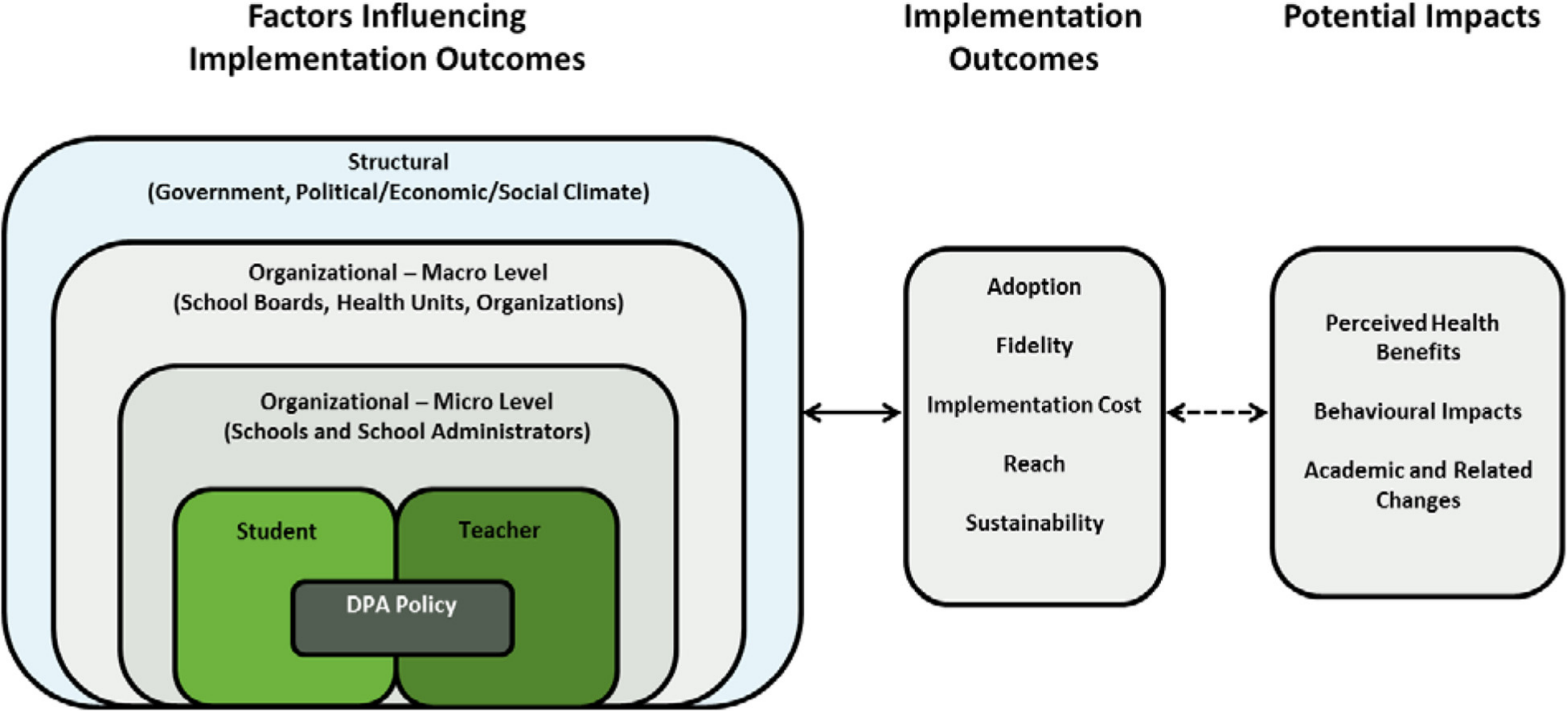
Conceptual framework of DPA studies, adapted from Chaudoir et al. [51]. The conceptual framework for this study is a derivative adapted from Chaudoir et al.’s “A multi-level framework predicting implementation outcomes” [45] and used under CC BY. The framework was adapted by further dividing organizational level factors into: 1) organizational-macro and 2) organizational-micro. A component demonstrating the potential benefits and impacts of implementation was also added. Using this framework, the study examined how factors at the organizational-micro and teacher levels may influence DPA implementation fidelity in Ontario elementary schools and classrooms

Dissemination and implementation research in health and related fields is an important component of policy and program evaluation [44]. For the related studies, a framework developed by Chaudoir and colleagues was adapted, depicting five levels of factors that influence implementation outcomes [45]. Specifically, the framework was adapted by dividing organizational level factors into: 1) organizational-macro and 2) organizational-micro. A component demonstrating the potential benefits and impacts of implementation was also added (Fig. 1).

This paper reports on the organizational-micro- (i.e., school and school administrator) and teacher-level factors that may influence DPA implementation in Ontario elementary schools and classrooms. The fidelity construct was the main implementation outcome assessed; fidelity being the extent to which the DPA policy is being implemented as originally intended [45]. More specifically, the research objectives addressed in the paper included: (1) Identifying the extent to which Ontario elementary *school administrators* perceive that DPA is being implemented in their school; (2) examining the association between DPA implementation and school-level characteristics, as reported by elementary *school administrators*; (3) identifying the extent to which Ontario elementary *school teachers* perceive that DPA is being implemented in their classroom and; (4) examining the association between DPA implementation and classroom-level characteristics, as reported by elementary *school teachers*. This paper provides an overview of study findings to contribute to both physical activity implementation research and its application.

## Methods

### Study design and approach

Two cross-sectional online surveys were conducted between March and June, 2014 – one among school administrators and the other with teachers, based on a representative random sample of Ontario elementary schools and classrooms. Ethical approval to conduct the study was received from PHO’s Ethics Review Board (ERB) in November 2013 (ERB ID: 2013-039.01). Administrators and teachers were required to provide informed consent in order to participate in the surveys.

### Study sample and recruitment

A stratified and nested random sampling strategy was used to identify the study sample. The sampling frame included all publicly funded Ontario elementary schools, from which a random sample of 532 schools was selected. The random sample of schools was designed to be representative of Ontario elementary schools based on the following characteristics: school board language (French versus English); school board type (public versus Catholic); school location (urban versus rural, based on postal code) [46]; and school enrolment size (small - up to 200 students, medium - 201-400, and large – more than 400) [47]. Specifically, the sample was stratified to reflect the proportion of Ontario schools within each category of the four characteristics. School administrators responded on behalf of their schools, in which one school administrator (principal or delegated vice-principal) responded per participating school.

Nested within schools, classrooms were randomly sampled from each of grades 3, 5 and 7, where possible (i.e., classrooms were only sampled from schools in which a school administrator had completed the survey). One homeroom teacher from each of the three grades responded on behalf of their respective classrooms. The three grades were selected to ensure representation from primary, junior and intermediate levels.

Participant recruitment occurred in three stages. First, approval was requested from school boards to conduct the study at the schools sampled within their board. Upon receiving school board approvals (30 of 40 school boards = 75.0 %), principals’ approvals to conduct the study within their schools were requested. One school administrator from each of the 228 schools that provided approval (228 of 532 schools = 42.9 %) was invited to participate in the survey through mailed information letters, followed by emailed invitations. Subsequently, similar mailed information letters and email invitations were sent to one teacher from each of grades 3, 5 and 7 (*n* = 508) in schools where a school administrator had completed a survey (*n* = 209). These teachers were randomly selected from a complete list of teaching staff provided by principals or school websites upon approval of the study.

To enhance recruitment and study quality, a stakeholder engagement strategy was deployed. Relevant stakeholders were identified based on their interest in, influence on and importance to the study. These key stakeholders included the Ontario government, our Study Advisory Committee, school board and school staff, principal and teacher associations, and public health units (PHUs) in Ontario. Areas of opportunity or concern for each stakeholder were then noted and this information was organized into a matrix. This matrix determined the relative level of engagement needed with each group of stakeholders and their potential influence on the study. Using this information, activities were developed for each group, including ongoing discussions with the EDU and sending question-and-answer sheets about the study to principal and teacher associations.

### Measures

Two similar instruments for the respective school administrator and teacher surveys were developed and informed by reviewing relevant items from existing survey instruments, the adapted Chaudoir framework [45], results from previous DPA studies [20], and DPA guidelines and other resource documents from the EDU [48–51].

The primary outcome was a measure of fidelity to DPA policy implementation [45]. Fidelity was further defined in the context of DPA implementation as six components, based on the six requirements of the DPA policy: 1) duration (minimum of 20 min); 2) frequency (each school day); 3) scheduling (during instructional time); 4) intensity (MVPA); 5) continuity (sustained physical activity for 20 min); and 6) inclusivity (including children with special needs). To measure the outcome, participants were asked to indicate whether DPA had been implemented at least once within their school (for school administrators) or classroom (for teachers) during the 2013-14 school year. If participants responded “Yes”, additional questions about DPA implementation within their school or classroom were asked, including six specific questions regarding fidelity to the six policy requirements described above.

The six fidelity questions asked participants to indicate the frequency (or number of days per week) that the DPA policy requirement in question was typically met or implemented in their school or classroom. Response options for each item ranged from 1–5: 1 = Never/1 day; 2 = Rarely/2 days; 3 = Sometimes/3 days; 4 = Often/4 days; and 5 = Always/5 days. The response option chosen for each individual item represented the fidelity score (out of five) for its corresponding DPA policy requirement. Internal consistency reliability (based on Cronbach’s alpha) of the six initial fidelity scale items was .90 at the school level and .98 at the classroom level, suggesting that internal consistency was high.

Using this scale, consisting of the six policy items, a composite score measuring overall implementation fidelity (out of 30) was calculated. This summed each participant’s fidelity score across the six items. Participants who answered “No” on the initial screening question (“Is DPA currently being implemented in your school/classroom?”), indicating DPA had not been implemented at least once in their school/classroom in 2013-14, were also included in analyses related to DPA implementation fidelity. In those cases, they received a composite score of zero for overall implementation fidelity.

For analysis and reporting purposes, the overall implementation fidelity scores were grouped into two categories. *Meets DPA policy requirements (scores 24–30)* included participants who indicated that DPA is currently being implemented in their school or classroom and *often or always* meets policy requirements (averaged a score of at least 4 across each of the six items). *Does not meet DPA policy requirements (scores 0–23.9*) included participants who indicated that DPA is *not* currently being implemented in their school or classroom (i.e., score of 0), or is currently being implemented but *never, rarely or sometimes* meets policy requirements (averaged a score of less than 4 across each of the six items).

When participants had missing or “I don’t know” responses to *less than four* questions related to implementation fidelity, the mean method of imputation was used to replace the missing values. Specifically, an average score was calculated using the scores of their responses to the other items that they completed in the scale. This value was then used in place of the missing values. Those with four or more missing or “I don’t know” responses were not included in the analyses related to overall implementation fidelity.

Several predictor variables were examined in relation to implementation fidelity, including: school sample characteristics (school board type, school board language, school location, school size); awareness of DPA policy requirements; perceptions of the DPA policy; scheduling and monitoring DPA; organization and instruction of DPA; use of DPA resources and supports; perceptions of barriers to implementing DPA; self-efficacy to carry out DPA activities; and personal characteristics of the respondents (gender, years of experience, prioritization of physical activity in daily personal life). School sample characteristics were obtained from the EDU, while personal characteristics were measured using multiple choice items on the survey instruments. Most other predictor variables were measured on the survey instruments using five-point Likert-type items. For analysis and reporting purposes, similar categories (e.g., agree/strongly agree) were combined in predictor variables with five response categories to make three categories.

**Table 1:** Descriptive analysis of participant and school characteristics

Characteristic	% School Administrators^a^ (n) ^b^	% Teachers^a^ (n) ^b^
Participant Characteristics		
Gender		
Female	67.9 (125)	71.9 (197)
Male	32.1 (59)	28.1 (77)
Year of experience in current role		
5 years or less	28.1 (52)	14.9 (41)
6 to 15 years	59.0 (109)	49.6 (137)
16 years or more	13.0 (24)	35.5 (98)
Level of HPE training		
University-level training	13.6 (25)	9.8 (27)
Other training (e.g., workshops, coaching certification)	10.3 (19)	9.4 (26)
Little to no training	76.2 (141)	80.8 (223)
Priority level of physical activity in daily life		
High	60.2 (112)	62.5 (172)
Moderate	28.5 (53)	30.9 (85)
Low	11.3 (21)	6.6 (18)
School Characteristics		
School board language		
English	94.3 (197)	94.1 (289)
French	5.7 (12)	5.9 (18)
School board type		
Public	71.8 (150)	68.4 (210)
Catholic	28.2 (59)	31.6 (97)
School location (based on postal code)		
Urban	74.2 (155)	72.0 (221)
Rural	25.8 (54)	28.0 (86)
School size		
Small (≤295 students)	50.7 (106)	50.5 (155)
Large (≥296 students)	49.3 (103)	49.5 (152)

^a^Percentage totals may not equal 100 % due to rounding

^b^Count totals (n) may not equal total sample (*n* = 209 for school administrators; *n* = 307 for teachers), and differ between variables, due to missing values

**Table 2:** Descriptive and independent bivariate analysis of predictors with overall implementation fidelity, school and classroom levels

Characteristic	Overall % school Administrators^a^ (n)^b^	Bivariate Associations at school level^c^		Overall % Teachers^a^ (n)^b^	Bivariate associations at classroom level^d^	
		OR (95 % CI)	*p*-value		OR (95 % CI)	*p*-value
Awareness of DPA policy requirements						
Overall awareness of policy requirements						
Aware of more than half	81.1 (163)	2.17 (1.05–4.46)	0.036	62.6 (189)	1.63 (1.00–2.65)	0.048
Aware of less than half	18.9 (38)	R	R	37.4 (113)	R	R
Scheduling and monitoring activities						
Scheduling in teachers’ timetables						
DPA is scheduled	66.5 (137)	4.39 (2.30–8.39)	<0.0005	67.0 (203)	3.38 (1.99–5.73)	<0.0005
DPA is not scheduled	29.6 (61)	R	R	33.0 (100)	R	R
I don’t know	3.9 (8)	--	--	--	--	--
Presence of school DPA monitoring procedure						
A procedure exists	25.2 (52)	4.73 (2.09–10.75)	<0.0005	10.5 (32)	4.89 (2.01–11.90)	0.001
A procedure does not exist	72.3 (149)	R	R	69.9 (214)	R	R
I don’t know	2.4 (5)	--	--	19.6 (60)	--	--
Organization of DPA delivery						
Type of DPA participation						
Several/all classes participate at the same time	8.9 (18)	3.75 (0.96–14.65)	0.057	10.2 (28)	1.54 (0.64–3.72)	0.332
Each class participates at separate times	65.8 (133)	1.21 (0.62–2.35)	0.581	48.5 (133)	1.10 (0.65–1.84)	0.724
Participation varies throughout the year	25.3 (51)	R	R	41.2 (113)	R	R
Individual instructing DPA						
Generalist teacher	83.7 (170)	0.46 (0.15–1.48)	0.195	75.9 (208)	0.95 (0.49–1.84)	0.881
Teacher with HPE specialization	8.4 (17)	R	R	16.8 (46)	R	R
Other	7.9 (16)	--	--	7.3 (20)	--	--
Perceived self-efficacy in carrying out DPA activities						
Confidence level in planning DPA						
High	65.2 (118)	1.18 (0.62–2.25)	0.613	62.3 (172)	5.36 (3.06–9.37)	<0.0005
Low-to-moderate	34.8 (63)	R	R	37.7 (104)	R	R
Confidence level in implementing DPA						
High	62.8 (113)	1.43 (0.76–2.69)	0.273	60.1 (161)	6.81 (3.87–11.97)	<0.0005
Low-to-moderate	37.2 (67)	R	R	39.9 (107)	R	R
Use of DPA resources and supports						
Frequency of using DPA resources						
Often or always	11.3 (23)	4.84 (1.54–15.18)	0.007	10.6 (32)	5.39 (2.15–13.48)	<0.0005
Occasionally	35.0 (71)	3.00 (1.56–5.78)	0.001	32.3 (98)	1.99 (1.18–3.36)	0.010
Never or rarely	53.7 (109)	R	R	57.1 (173)	R	R
Frequency of using DPA supports						
Often or always	8.7 (18)	13.54 (1.74–105.50)	0.013	5.3 (16)	6.68 (1.79–24.86)	0.005
Occasionally	39.1 (81)	1.35 (0.75–2.45)	0.315	25.1 (76)	3.91 (2.17–7.02)	<0.0005
Never or rarely	52.2 (108)	R	R	69.7 (211)	R	R
Frequency of communicating with public health units regarding DPA						
Often or always	6.8 (14)	1.34 (0.43–4.20)	0.616	1.0 (3)	--	--
Occasionally	23.3 (48)	1.95 (0.95–4.00)	0.070	4.3 (13)	2.39 (0.70–8.14)	0.164
Never or rarely	69.9 (144)	R	R	94.7 (286)	R	R
Perceptions of DPA policy						
Clear and easy to understand						
Agree/strongly agree	85.6 (178)	1.40 (0.36–5.40)	0.625	82.9 (247)	1.98 (0.62–6.31)	0.245
Neutral	10.1 (21)	0.73 (0.15–3.49)	0.691	12.4 (37)	1.24 (0.33–4.62)	0.746
Disagree/strongly disagree	4.3 (9)	R	R	4.7 (14)	R	R
Realistic and achievable						
Agree/strongly agree	56.0 (117)	3.29 (1.68–6.44)	0.001	43.0 (129)	8.61 (4.85–15.27)	<0.0005
Neutral	18.2 (54)	1.89 (0.80–4.43)	0.145	16.0 (48)	2.20 (1.089–4.46)	0.028
Disagree/strongly disagree	25.8 (38)	R	R	41.0 (123)	R	R
Equally important as other school curriculum requirements						
Agree/strongly agree	75.4 (156)	1.99 (0.81–4.89)	0.135	58.4 (175)	3.13 (1.72–5.68)	<0.0005
Neutral	14.0 (29)	2.28 (0.73–7.10)	0.155	16.7 (50)	1.37 (0.63–2.98)	0.427
Disagree/strongly disagree	10.6 (22)	R	R	25.0 (75)	R	R
Impact on students’ physical well-being						
Somewhat positive/very positive	93.3 (194)	1.22 (0.41–3.67)	0.720	91.8 (279)	1.54 (0.65–3.62)	0.325
Neither positive nor negative	6.7 (14)	R	R	8.2 (25)	R	R
Somewhat negative/very negative	0.0 (0)	--	--	0.0 (0)	--	--
Impact on students’ emotional well-being						
Somewhat positive/very positive	90.4 (188)	1.08 (0.42–2.77)	0.873	89.1 (269)	1.37 (0.64–2.95)	0.419
Neither positive nor negative	9.6 (20)	R	R	10.6 (32)	R	R
Somewhat negative/very negative	0.0 (0)	--	--	0.3 (1)	--	--
Impact on students’ academic outcomes						
Somewhat positive/very positive	82.9 (170)	1.32 (0.62–2.77)	0.471	71.6 (220)	1.46 (0.84–2.54)	0.183
Neither positive nor negative	16.6 (34)	R	R	24.1 (71)	R	R
Somewhat negative/very negative	0.5 (1)	--	--	1.4 (4)	--	--
Impact on student conduct						
Somewhat positive/very positive	87.3 (178)	1.45 (0.61–3.41)	0.401	78.7 (236)	1.15 (0.64–2.09)	0.636
Neither positive nor negative	11.8 (24)	R	R	19.3 (58)	R	R
Somewhat negative/very negative	1.0 (2)	--	--	2.0 (6)	--	--
Impact on students’ social well-being						
Somewhat positive/very positive	82.4 (168)	2.48 (1.18–5.21)	0.016	75.0 (225)	1.40 (0.81–2.41)	0.231
Neither positive nor negative	17.2 (35)	R	R	24.3 (73)	R	R
Somewhat negative/very negative	0.5 (1)	--	--	0.7 (2)	--	--
Impact on the development of physical activity habits						
Somewhat positive/very positive	87.1 (175)	1.34 (0.57–3.12)	0.501	80.6 (241)	1.58 (0.86–2.91)	0.142
Neither positive nor negative	12.4 (25)	R	R	19.4 (58)	R	R
Somewhat negative/very negative	0.5 (1)	--	--	0.0 (0)	--	--

^a^Percentage totals may not equal 100 % due to rounding

^b^Count totals (n) may not equal total sample (*n* = 209 for school administrators; *n* = 307 for teachers), and differ between variables, due to missing values

^c^Bivariate analysis at school level conducted using logistic regression

^d^Bivariate analysis at classroom level conducted using generalized linear mixed models to adjust for school-level clustering effects

^R^Reference category

^--^Categories with low counts (overall frequency ≤ 2.0 %) omitted from bivariate analysis

**Table 3:** Descriptive and independent bivariate analysis of barriers to DPA with overall implementation fidelity, school and classroom levels

Barrier	Overall % school administrators^a^ (n)^b^	Bivariate associations at school level^c^		Overall % Teachers^a^ (n)^b^	Bivariate associations at classroom level^d^	
		OR (95 % CI)	*p*-value		OR (95 % CI)	*p*-value
Competing curriculum priorities						
Disagree/strongly disagree	15.1 (30)	3.63 (1.31–10.02)	0.013	12.2 (35)	5.54 (2.29–13.38)	<0.0005
Neutral	8.5 (17)	2.46 (0.77–7.88)	0.131	9.4 (27)	2.71 (1.14–6.41)	0.024
Agree/strongly agree	76.4 (152)	R	R	78.5 (226)	R	R
Lack of time						
Disagree/strongly disagree	26.7 (54)	5.75 (2.51–13.19)	<0.0005	14.7 (43)	7.23 (3.02–17.31)	<0.0005
Neutral	11.9 (24)	2.00 (0.80–5.02)	0.140	6.5 (19)	2.58 (0.95–7.03)	0.064
Agree/strongly agree	61.4 (124)	R	R	78.8 (230)	R	R
Lack of teacher readiness						
Disagree/strongly disagree	32.8 (66)	4.22 (2.00–8.90)	<0.0005	38.2 (108)	2.16 (1.25–3.73)	0.006
Neutral	19.9 (40)	1.034 (0.49–2.18)	0.925	20.8 (59)	0.97 (0.50–1.86)	0.923
Agree/strongly agree	47.3 (95)	R	R	41.0 (116)	R	R
Lack of space						
Disagree/strongly disagree	58.4 (118)	2.43 (1.29–4.58)	0.006	23.8 (69)	3.15 (1.72–5.74)	<0.0005
Neutral	10.4 (21)	1.30 (0.47–3.59)	0.608	13.1 (38)	1.72 (0.83–3.57)	0.146
Agree/strongly agree	31.2 (63)	R	R	63.1 (183)	R	R
Bad weather						
Disagree/strongly disagree	49.8 (101)	2.13 (1.11–4.09)	0.023	38.7 (111)	3.03 (1.70–5.42)	<0.0005
Neutral	19.7 (40)	2.07 (0.90–4.76)	0.088	25.8 (74)	1.17 (0.62–2.21)	0.629
Agree/strongly agree	30.5 (62)	R	R	35.5 (102)	R	R
Students’ reluctance to participate						
Disagree/strongly disagree	55.0 (111)	0.93 (0.48–1.82)	0.833	51.4 (146)	1.56 (0.90–2.71)	0.113
Neutral	17.8 (36)	0.88 (0.37–2.11)	0.779	17.3 (49)	0.87 (0.42–1.82)	0.711
Agree/strongly agree	27.2 (55)	R	R	31.3 (89)	R	R
Lack of equipment						
Disagree/strongly disagree	68.0 (138)	1.82 (0.85–3.87)	0.122	41.4 (120)	2.39 (1.41–4.04)	0.001
Neutral	14.8 (30)	1.64 (0.60–4.48)	0.338	15.9 (46)	1.09 (0.54–2.21)	0.814
Agree/strongly agree	17.2 (35)	R	R	42.8 (124)	R	R
Lack of resources						
Disagree/strongly disagree	59.2 (119)	1.85 (0.83–4.12)	0.131	38.7 (111)	3.73 (2.08–6.69)	<0.0005
Neutral	25.4 (51)	1.36 (0.55–3.36)	0.507	23.7 (68)	0.94 (0.49–1.81)	0.855
Agree/strongly agree	15.4 (31)	R	R	37.6 (108)	R	R
Lack of school board support						
Disagree/strongly disagree	48.0 (96)	2.11 (0.89–4.99)	0.091	31.7 (91)	4.19 (2.24–7.83)	<0.0005
Neutral	38.0 (76)	1.02 (0.43–2.44)	0.965	34.1 (98)	1.66 (0.92–2.98)	0.093
Agree/strongly agree	14.0 (28)	R	R	34.1 (98)	R	R
Lack of amenities						
Disagree/strongly disagree	72.0 (144)	1.70 (0.73–3.94)	0.217	56.3 (162)	3.22 (1.67–6.22)	0.001
Neutral	15.0 (30)	1.64 (0.56 – 4.79)	0.369	23.3 (67)	1.62 (0.76–3.48)	0.214
Agree/strongly agree	13.0 (26)	R	R	20.5 (59)	R	R
Lack of parent/guardian support						
Disagree/strongly disagree	60.2 (118)	1.95 (0.75–5.07)	0.171	50.7 (142)	1.28 (0.64–2.55)	0.483
Neutral	29.6 (58)	1.24 (0.45–2.45)	0.680	32.9 (92)	0.64 (0.30–1.33)	0.227
Agree/strongly agree	10.2 (20)	R	R	16.4 (46)	R	R

^a^Percentage totals may not equal 100 % due to rounding

^b^Count totals (n) may not equal total sample (*n* = 209 for school administrators; *n* = 307 for teachers), and differ between variables, due to missing values

^c^Bivariate analysis at school level conducted using logistic regression

^d^Bivariate analysis at classroom level conducted using generalized linear mixed models to adjust for school-level clustering effects

^R^Reference category

### Data collection

Dillman’s Tailored Design Method [52], which has been shown to enhance participation in surveys administered by mail, internet, and mixed methods [35, 52–54] was applied for data collection. To encourage study participation, a number of procedures for contacting potential participants (i.e., email, mail, and phone) were used. Participants were also provided a $10 gift card to a bookstore in the original letter inviting them to participate.

Both the school administrator and teacher surveys were conducted using FluidSurveys [55]. Each survey instrument consisted primarily of closed-ended questions (e.g., multiple choice and Likert-scale questions) and was designed to take approximately 15 min to complete.

School administrator surveys were administered in multiple waves between February and April 2014, while teacher surveys were administered in waves from April to June 2014. Both school administrator and teacher surveys remained open for 5 weeks, from the first day in which it was sent to each wave. Reminder letters were sent (by email or letter) to participants at weeks two and four of each wave.

School administrators and teachers received similar surveys. They were asked to respond to the same questions regarding DPA implementation; school administrators from a school-level perspective and teachers from a classroom-level perspective. The school administrator survey also included additional questions regarding DPA planning at the school level. Respondents could choose to complete the survey instruments in either official language, English or French.

### Data analysis

School administrator and teacher survey data were imported into IBM SPSS version 21 (SPSS Inc., Chicago, IL, USA) for analysis. Univariate analyses captured descriptive characteristics of the sample and determined the distribution of responses for the outcome and selected predictor variables. Binary logistic regression was used with school administrator survey data to examine the relationship between various predictors and implementation fidelity at the school level. For teacher survey data, generalized linear mixed models (GLMM) were used to adjust for school-level clustering effects, when examining the relationship between predictors and implementation fidelity at the classroom level.

Significant *p*-values (*p* < 0.05) from the logistic regressions and GLMMs were reported to demonstrate the association between predictor and outcome variables at the school and classroom levels, respectively. All findings were reported at an aggregate (provincial) level.

## Results

### Participant and school board characteristics

Of the 532 school administrators invited to participate in the study, 209 responded to the school administrator survey for a response rate of 39.3 %. Of these respondents, the majority were female (67.9 %), and more than half (59.0 %) have had 6–15 years of experience in their role. Most (76.2 %) reported having little or no training in HPE, although 60.2 % said physical activity was a high priority in their daily life. Of the 508 teachers invited to participate in the teacher survey, 307 responded, yielding a response rate of 60.4 %. Most were female (71.9 %), and about half (49.6 %) have had 6–15 years of experience in teaching. While most (80.8 %) reported having little or no previous PE training, just under two-thirds (62.5 %) reported physical activity as being a high priority in their personal lives. The distribution of participant and school characteristics, for both school administrator and teacher responses, is shown in Table 1. The distribution of the final analytic sample was found to be similar to the distribution of all publicly funded elementary schools in the province, based on school board language, school board type, school location, and school size.

### Implementation fidelity

**Fig. 2:**
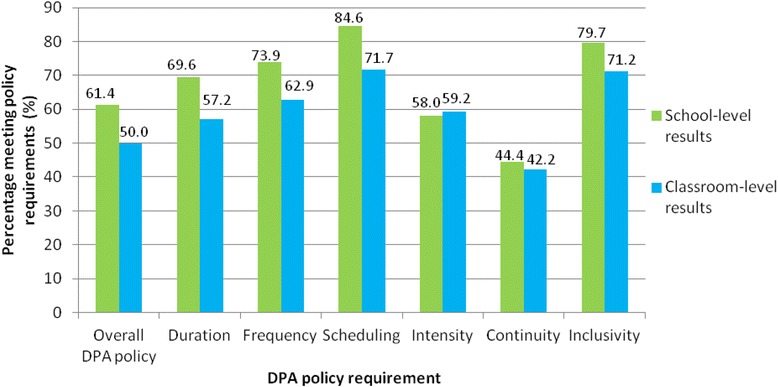
School- and classroom-level implementation fidelity to overall DPA policy and individual policy requirements

Findings from the surveys of administrators and teachers indicated incomplete and inconsistent DPA implementation. Notably, a higher percentage of school administrators (61.4 %) indicated that their school was meeting the DPA policy requirement than teachers at the classroom level (50 %). These apparent differences occurred overall and for most of the specific components of the policy (Fig. 2). Reported implementation fidelity, at both the school and classroom levels, was lower for both intensity (MVPA) and continuity (sustained physical activity), compared to other components. At the school level, 58.0 % of school administrators reported meeting the intensity component, while 44.4 % reported meeting the continuity component. Similarly, 59.2 % and 42.2 % of teachers reported meeting these two components, respectively, at the classroom level. In addition, 57.2 % of teachers reported meeting the duration component.

Upon further assessment of implementation fidelity in relation to potential influencing factors (predictors), several significant associations were found. Table 2 presents the overall distribution of school administrator and teacher responses for each predictor explored and their associations with overall implementation fidelity at the school and classroom levels.

### Participant and school characteristics and implementation fidelity

The number of years as a school administrator was significantly related to implementation fidelity at the school level (*p* = 0.017). Specifically, administrators who have worked more than 16 years were more likely to report meeting the guidelines than those who have worked less than 5 years (OR = 5.07, 95 % CI = 1.34–19.24) (not shown in Table 2). However, the strength of this relationship should be interpreted with caution due to the large confidence intervals. At the classroom level, grade level was significantly related to implementation fidelity (*p* = 0.043). Specifically, grade 5 teachers were more likely to report meeting the guidelines compared to grade 3 teachers (OR = 1.82, CI = 1.02–3.24) (not shown in Table 2). Significant differences in overall implementation fidelity at the school level (*p* = 0.012) were also found based on board type, with higher fidelity in public schools compared to Catholic schools (OR = 2.22, 95 % CI = 1.19–4.14).

### Awareness of DPA and implementation fidelity

Among school administrators, 81.1 % reported awareness of more than half of the DPA policy components, while awareness of more than half was reported by 62.6 % of teachers. Awareness was significantly related to implementation fidelity at both the school (*p* = 0.036) and classroom (*p* = 0.048) levels. Administrators with higher awareness of the DPA policy requirements were more likely to report implementation fidelity in their schools compared to those with lower awareness (OR = 2.17, 95 % CI = 1.05–4.46). This pattern was also found for teachers reporting at the classroom level (OR = 1.63, 95 % CI = 1.00–2.65). This pattern was replicated, in several instances, when comparing awareness with implementation fidelity for specific policy components (for example, awareness of the duration component in relation to implementation fidelity for that specific component).

### Scheduling and monitoring activities and implementation fidelity

Approximately two-thirds of school administrators (66.5 %) and teachers (67.0 %) reported that DPA was scheduled in teachers’ timetables. A key finding was the significant relationship between scheduling DPA in teachers’ timetables and overall implementation fidelity at both school (*p* < 0.0005) and classroom (*p* < 0.0005) levels. Both school administrators (OR = 4.39, 95 % CI = 2.30–8.39) and teachers (OR = 3.38, 95 % CI = 1.99–5.73) were more likely to report meeting the policy requirements if DPA was scheduled in teachers’ timetables.

Another important finding was the level of DPA monitoring in schools and its significant relationship with overall implementation fidelity at both school (*p* < 0.0005) and classroom (*p* = 0.001) levels. Based on survey findings, 72.3 % of administrators and 69.9 % of teachers reported no monitoring procedures for DPA at the school level. However, schools having a DPA monitoring procedure were more likely to have implementation fidelity at the school (OR = 4.73, 95 % CI = 2.09–10.75) and classroom (OR = 4.89, 95 % CI = 2.01–11.90) levels, compared to schools that did not have such a procedure.

### Organization of DPA delivery and implementation fidelity

The study findings indicated non-significant associations between form of DPA delivery and overall implementation fidelity.

### Perceived self-efficacy and implementation fidelity

Teachers expressing confidence in successfully planning (62.3 %) and implementing (60.1 %) DPA were more likely (OR = 5.36, 95 % CI = 3.06–9.37 for planning; and OR = 6.81, 95 % CI = 3.87–11.97 for implementing) to report implementation fidelity in their classroom than teachers expressing low or moderate confidence (*p* < 0.0005). This relationship was not borne out for school administrators.

### Use of DPA resources and supports and implementation fidelity

Both school administrators and classroom teachers reported very infrequent use of DPA resources (i.e., learning tools) and supports (i.e., individuals or organizations). Among school administrators, 11.3 % and 8.7 % reported often or always using DPA resources and supports, respectively. Similarly, 10.6 % and 5.3 % of teachers reported often or always using DPA resources and supports, respectively. Use of DPA resources and supports was significantly related to implementation fidelity at the school and classroom levels. A consistent pattern was that those using DPA resources and supports often or always (or even occasionally in some cases) were more likely to report implementation fidelity at both the school and classroom levels (Table 2). However, it is important to note that, while the odds ratios are relatively high, the strengths of these relationships may be weaker since the 95 % confidence intervals are quite large. With respect to communicating with public health units (PHUs) about DPA, 69.9 % of administrators and 94.7 % of classroom teachers indicated that they never or rarely do so. Also, analysis indicated no significant relationships between communicating with PHUs and DPA implementation fidelity at the school or classroom level.

### Perceptions of the DPA policy and implementation fidelity

Only 56.0 % of administrators and 43.0 % of teachers perceived the DPA policy to be realistic and achievable. This perception was significantly related to implementation fidelity at both the school (*p* = 0.001) and classroom (*p* < 0.0005) levels. Specifically, those agreeing/strongly agreeing that the policy is realistic and achievable were more likely to report greater implementation fidelity at the school (OR = 3.29, 95 % CI = 1.68–6.44) and classroom level (OR = 8.61, 95 % CI = 4.85–15.27) than those disagreeing/strongly disagreeing that the policy is realistic and achievable.

DPA was considered to be equally important as other curriculum requirements, according to 75.4 % of school administrators, although this was not significantly related to school-level implementation fidelity. However, a lower percentage (58.4 %) of classroom teachers agreed or strongly agreed with this statement, and this was found to be significantly related to implementation fidelity at the classroom level (*p* < 0.0005). Specifically, teachers who agreed or strongly agreed that DPA is equally important as other curriculum requirements were more likely to report implementation fidelity at the classroom level (OR = 3.13, 95 % CI = 1.72–5.68).

Both administrators and teachers perceived that DPA is associated with a number of benefits, including improvements in students’ physical, social, and emotional well-being, academic outcomes, conduct, and physical activity habits. However, perceptions about these benefits, with one exception (impact on social well-being), were not significantly related to implementation fidelity at the school and classroom levels.

### Perceived barriers to DPA implementation

Many school administrators (76.4 %) and classroom teachers (78.5 %) agreed or strongly agreed that *competing curriculum priorities* were barriers to policy implementation in schools and classrooms respectively. This pattern was also apparent in the case of *lack of time*, with 61.4 % of administrators and 78.8 % of teachers agreeing/strongly agreeing that this was a barrier to implementation. However, several administrators (47.3 %) agreed/strongly agree that *lack of teacher readiness* was an important factor, while 63.1 % of classroom teachers agreed/strongly agreed that *lack of space* was a prominent barrier (Table 3).

In addition, many of the barriers assessed were significantly related to meeting the DPA policy requirement, especially at the classroom level (Table 3). The dominant pattern was that those disagreeing or strongly disagreeing with the prominence of a barrier were more likely to report meeting the DPA requirement compared to those agreeing/strongly agreeing with the prominence of a barrier. For example, administrators (OR = 3.63, 95 % CI = 1.31–10.02) and teachers (OR = 5.54, 95 % CI = 2.29–13.38) who disagreed/strongly disagreed that *competing curriculum priorities* were a barrier to policy implementation were more likely to report implementation fidelity at the school and classroom level respectively. Similarly, administrators (OR = 5.75, 95 % CI = 2.51–13.19) and teachers (OR = 7.23, 95 % CI 3.02–17.31) who disagreed/strongly disagreed that *lack of time* was a barrier to policy implementation were more likely to report implementation fidelity at the school and classroom levels respectively. These general patterns were replicated for both administrators and teachers in the case of several additional barriers: *lack of teacher readiness*, *lack of space*, and *bad weather*. In addition, the pattern was replicated specifically for teachers in the case of: *lack of equipment*, *lack of resources*, *lack of school board support*, and *lack of amenities*.

## Discussion

While full implementation fidelity is an expectation of the Ontario Ministry of Education (EDU) and a curriculum requirement of all publicly funded school boards in the province, findings from this study indicated incomplete and inconsistent implementation. In addition, a lower percentage of teachers reported implementation fidelity in their classrooms compared to administrators reporting on a school level. Besides being based on separate surveys, the findings may have also reflected the respective frames of reference for these two groups. Teachers, who are typically responsible for implementing DPA, had a more specific understanding of the extent to which they were implementing the components of the policy in their classroom, while administrators provided a more general assessment of DPA implementation for the school overall.

Another important finding with respect to implementation was that two components of implementation fidelity (intensity and continuity) were reported by administrators and teachers as lower than most other components. This may relate to an uneven understanding of the policy requirements for these components, due partly to ambiguity in the source documents provided to administrators and teachers. Specifically, PPM No. 138 states that 20 min of sustained MVPA is to be provided to students [19], while guidance documents indicate that time for a warm-up and cool-down are *included* in the 20-min DPA session [48–51]. Also, for practical reasons, it is believed that some teachers offer shorter bouts of activity during the day. Stone and colleagues indicated such a perspective (that students are more likely to complete a number of shorter sessions than 20 min of sustained MVPA) after finding that no students were meeting the current requirements in their study of a number of Toronto elementary schools [42]. However, it is unclear if this assumption is correct, or whether schools can meet the 20 min of sustained activity at MVPA intensity under more favourable scheduling and logistical conditions.

Consistent with the adapted Chaudoir framework [45] and Social Ecological Model [25], our study findings indicated a number of predictors of implementation fidelity in schools and classrooms at various social ecological levels. While these can be categorized overall as organizational-micro focus (Fig. 1), several predictors can also be considered individual-level and organizational/system-level factors influencing DPA implementation.

### Individual-level predictors

Not surprisingly, *awareness* of the policy was significantly related to implementation fidelity. Yet, it is important to acknowledge that awareness of policy requirements, by itself, may be a necessary but not sufficient factor related to implementation fidelity. Additional factors were also found to be related to implementation fidelity, as described below.

According to Bandura’s concept of *self-efficacy* (as part of Social Cognitive Theory), individuals who have confidence in taking action, or in overcoming barriers to take action, are more likely to implement the related behaviour [56]. Findings from the study supported this claim in the case of classroom teachers. Teacher self-efficacy has been shown to be an important predictor of physical activity policy/curriculum implementation in previous studies [27, 30, 39].

Findings from the study also confirm the importance of some additional perceptions that administrators and teachers had, which were significantly related to implementation fidelity. One explanation for the lower *perceived priority of DPA* among teachers, as compared to administrators, is that teachers may experience more direct constraints on what can be accomplished in their classroom. For example, Patton’s study of DPA implementation found that administrators very much favoured DPA programs while teachers held a more instrumental view, maintaining that implementation lacked logistical supports [38]. In any case, it is interesting to note that study findings revealed that implementation fidelity was higher in classrooms where teachers agreed with the notion that DPA was equally important as other curriculum requirements, while this was not a significant relationship among school administrators.

As mentioned earlier, administrators and teachers perceived that DPA is associated with a number of *benefits*. Conceptually, these perceived benefits were considered to be potential facilitating factors, but findings from the analysis indicated that they were not significantly associated with implementation fidelity. This indicated that variability in implementation fidelity may be more related to other factors (e.g., awareness of DPA requirements, scheduling, monitoring and barriers) than to perceptions about DPA’s benefits to student well-being.

### Organizational/system-level predictors

In developing the study and measurement instrument, *resources and supports* were conceptualized as facilitating factors in relation to implementation fidelity, as previous studies have indicated their importance [26–28]. However, the findings indicated that they need to be considered as potential rather than actual facilitators since administrators and teachers reported infrequent use of these. It is unclear whether this was due to a lack of (or lack of awareness of) available resources and supports. In the specific case of resources, perhaps they were not current, or newer resources and learning opportunities were offered on potentially more prominent topics. In any case, the significant association of using resources and supports with implementation fidelity indicates that they could have an important potential role in achieving higher levels of implementation.

A key finding was that *scheduling and monitoring* were found to be important predictors of DPA implementation fidelity in both schools and classrooms. In, particular, despite low levels of reported monitoring at the school and classroom levels, it was a significant predictor of implementation fidelity. In a prior study of DPA’s development and implementation in Ontario [20], key informants indicated that regular monitoring was a central factor in assessing DPA policy implementation and effectiveness. An important tool for examining policy status, regular monitoring is considered crucial to assessing accountability [14]. It can also contribute to ongoing information for those responsible for implementing DPA, such as school boards, school administrators and classroom teachers.

Some of the most consistent findings were related to *perceived barriers* to DPA implementation in schools and classrooms such as: competing curriculum priorities; lack of time; lack of teacher readiness; and lack of space. Moreover, perceived barriers were negatively associated with implementation fidelity. A number of similar perceived barriers and related factors, described earlier in this paper, have also been shown to be associated with physical activity policy/curriculum implementation in previous studies [26–28, 30, 32, 38].

### Study limitations

Research in school settings presents a number of challenges and this study was no exception. While DPA is a policy requirement for school boards and schools, research on this topic is not always seen as a high priority among these groups. School boards, school administrators, and classroom teachers are extremely busy and face many requests for research and other projects to consider in addition to their core responsibilities. In the recruitment phase of this study, some administrators expressed hesitation about having their school participate, citing concerns about competing priorities and overburdening staff.

Given these systemic challenges, the research team designed and conducted a study intended to enhance participation and produce the best data possible. In order for study findings to be considered potentially generalizable to the underlying distribution of publicly funded schools in Ontario, a stratified random sample design was used for the school surveys. The final analytic sample compares quite favourably with the underlying distribution of publicly funded elementary schools in the province, providing some evidence that the findings may be representative.

The school-level response rate, based on survey completions by school administrators, was similar to, or better than, response rates achieved in a number of other online school surveys [26, 57]. However, it limits the extent to which findings can be generalized regarding implementation fidelity. It could be argued, for example, that non-participating schools from the sample drawn would be less likely than participating schools to reflect higher DPA implementation fidelity. Thus, findings may be overestimates of implementation fidelity. This would, in effect, underestimate the extent of the implementation problem (although this was not determined).

The school-level response rate resulted in lower analytic sample sizes than planned at the school and classroom levels. This influenced the statistical significance of associations between a number of factors and overall implementation fidelity. Study findings reflect a large number of significant associations between factors (e.g., awareness, scheduling, monitoring, barriers) and overall implementation fidelity. Still, some other relationships may have reached significance with a larger analytic sample size.

To address the challenges of recruiting school administrators and teachers, a number of strategies were applied, including proactive use of the Dillman approach for online surveys [52]. For example, small gifts of appreciation were provided during recruitment, and reminder messages were sent using different modes of communication (e-mail, regular mail, courier, and personal telephone calls). These approaches, along with a stakeholder engagement strategy, contributed to some extent in increasing survey participation.

An additional limitation was that the study was based on self-reported data from school administrators and classroom teachers. More specifically, measures of implementation fidelity were not validated with information from external sources. However, based on a review of existing survey measures, a set of parallel questions was also included to assess information similar to the DPA policy requirements. For example, the survey contained separate questions on the number of minutes of MVPA in DPA sessions. Within the scope of the current study, it was not feasible to have direct observation of DPA activities, objectively measure components such as the duration and intensity of DPA activities, or obtain classroom timetables.

It is assumed that, for the most part, participating school administrators and classroom teachers provided informed and accurate information and assessments. They should be very familiar with these issues. However, it is possible that some participating teachers, for example, may not themselves be delivering DPA for their students. Instead, DPA may be delivered by a PE teacher or someone else. In such cases, the classroom teacher participating in the survey may not have been the most informed about the specific information requested regarding DPA for students in their classroom.

Due to the cross-sectional design of the surveys, the relationships between predictors and implementation fidelity should not be interpreted as causal. Finally, the findings reported in this overview paper were based on descriptive and independent bivariate analyses. Future analyses to be conducted by the research team, once data from the two surveys are merged, will examine these associations together using advanced regression models, including multi-level analysis.

## Conclusions

The larger context of this study is the extensive research on jurisdictional policies and their relationship to physical activity and physical education opportunities and participation. Much of this important work has focussed on state-level policies around physical education requirements and systemic barriers (such as exemptions) to implementing these in the United States [58, 59]. We believe the current study contributes to the further development of similar studies in Canada.

This is the first provincial level study of DPA policy implementation fidelity and its predictors in Ontario. The study assesses DPA implementation at both the school and classroom levels. Also, a fidelity score, based on the six required components of the policy, was developed and used to assess implementation in the study. Finally, the study presents an adapted conceptual framework to situate factors related to DPA implementation fidelity.

The study suggests a number of important implications for policy, practice, and further research. Most importantly, the findings confirm that DPA is not being implemented uniformly in Ontario elementary and middle schools. Furthermore, a number of factors significantly associated with implementation fidelity were found, such as awareness of the policy requirements, scheduling, monitoring, teacher self-efficacy, and barriers at both an individual and system level.

These findings provided evidence to inform potential government action at the policy and program level. A number of evidence-informed recommendations have been submitted to the Ontario Ministry of Education dealing with such issues as accountability for monitoring DPA implementation, development of innovative approaches to enhance teacher self-efficacy through continued teacher training, and development of creative resources. The findings also suggest the need for further analysis and additional evaluation research, to both monitor policy implementation and assess the impacts and outcomes of DPA going forward. Finally, the study prompts the need to consider more fundamental policy issues concerning optimal approaches for providing structured opportunities for daily school-based physical activity.

## Abbreviations

DPA, daily physical activity; EDU, Ontario Ministry of Education; GLMM, generalized linear mixed models; HPE, health and physical education; MVPA, moderate-to-vigorous physical activity; PE, physical education; PHO, Public Health Ontario; PHU, public health unit; PPM, policy/program memorandum
